# Improving Adaptive and Memory Immune Responses of an HIV/AIDS Vaccine Candidate MVA-B by Deletion of Vaccinia Virus Genes (C6L and K7R) Blocking Interferon Signaling Pathways

**DOI:** 10.1371/journal.pone.0066894

**Published:** 2013-06-27

**Authors:** Juan García-Arriaza, Pilar Arnáez, Carmen E. Gómez, Carlos Óscar S. Sorzano, Mariano Esteban

**Affiliations:** 1 Department of Molecular and Cellular Biology, Centro Nacional de Biotecnología, Consejo Superior de Investigaciones Científicas (CSIC), Madrid, Spain; 2 Biocomputing Unit, Centro Nacional de Biotecnología, Consejo Superior de Investigaciones Científicas (CSIC), Madrid, Spain; University of Massachusetts Medical Center, United States of America

## Abstract

Poxvirus vector Modified Vaccinia Virus Ankara (MVA) expressing HIV-1 Env, Gag, Pol and Nef antigens from clade B (termed MVA-B) is a promising HIV/AIDS vaccine candidate, as confirmed from results obtained in a prophylactic phase I clinical trial in humans. To improve the immunogenicity elicited by MVA-B, we have generated and characterized the innate immune sensing and the *in vivo* immunogenicity profile of a vector with a double deletion in two vaccinia virus (VACV) genes (*C6L* and *K7R*) coding for inhibitors of interferon (IFN) signaling pathways. The innate immune signals elicited by MVA-B deletion mutants (MVA-B ΔC6L and MVA-B ΔC6L/K7R) in human macrophages and monocyte-derived dendritic cells (moDCs) showed an up-regulation of the expression of IFN-β, IFN-α/β-inducible genes, TNF-α, and other cytokines and chemokines. A DNA prime/MVA boost immunization protocol in mice revealed that these MVA-B deletion mutants were able to improve the magnitude and quality of HIV-1-specific CD4^+^ and CD8^+^ T cell adaptive and memory immune responses, which were mostly mediated by CD8^+^ T cells of an effector phenotype, with MVA-B ΔC6L/K7R being the most immunogenic virus recombinant. CD4^+^ T cell responses were mainly directed against Env, while GPN-specific CD8^+^ T cell responses were induced preferentially by the MVA-B deletion mutants. Furthermore, antibody levels to Env in the memory phase were slightly enhanced by the MVA-B deletion mutants compared to the parental MVA-B. These findings revealed that double deletion of VACV genes that act blocking intracellularly the IFN signaling pathway confers an immunological benefit, inducing innate immune responses and increases in the magnitude, quality and durability of the HIV-1-specific T cell immune responses. Our observations highlighted the immunomodulatory role of the VACV genes *C6L* and *K7R*, and that targeting common pathways, like IRF3/IFN-β signaling, could be a general strategy to improve the immunogenicity of poxvirus-based vaccine candidates.

## Introduction

The development of an effective HIV/AIDS vaccine is one of the main objectives worldwide in the pursuit of an eradication of this pandemic disease, prevent HIV-1 infection and limit viral transmission. Among the different approaches, improved poxvirus vectors should be considered as components of a future effective HIV/AIDS vaccine [1], [2], based on the results obtained with a combination of the recombinant poxvirus vector ALVAC and the HIV-1 gp120 protein, which showed 31% protection against HIV-1 infection in a phase III clinical trial (RV144) in Thailand [3].

Among poxviruses, the highly attenuated vaccinia virus (VACV) strain Modified Vaccinia Virus Ankara (MVA) is one of the most promising vectors to be used as an effective vaccine against HIV-1 [1], [2], [4]. MVA is a safe vector and despite its limited replication in human and most mammalian cell types, provides a high level of gene expression and triggers antigen-specific immune responses when delivered to animals and humans [1], [2], [4]. Many MVA recombinants expressing different HIV-1 antigens have been generated worldwide, and tested in different animal models and in several clinical trials in humans [1], [5], [6], [7], [8], [9], [10], [11], [12], [13], showing that they are safe, able to induce high levels of expression of HIV-1 antigens, and triggered immune responses to HIV-1 antigens. In particular, we have constructed a recombinant MVA expressing Env, as monomeric gp120, and the codon-optimized polyprotein Gag-Pol-Nef (GPN) of HIV-1 from clade B (referred as MVA-B), that in mice induced strong, broad, polyfunctional and durable HIV-1 specific CD4^+^ and CD8^+^ T cell immune responses (with a bias for CD8^+^) [14], [15], [16], [17]. In macaques, a similar MVA construct expressing Env (gp120 from SHIV_89.6P_) and GPN (from SIV_mac239_) showed strong specific HIV/SIV CD4^+^ and CD8^+^ T cell immune responses, and trigger protection following challenge with pathogenic strain SHIV_89.6P_ [18]. Based on all these previous results, MVA-B entered in a phase I clinical trial (RISVAC02) in 30 human healthy volunteers in Spain and the results showed that MVA-B was safe, well tolerated and highly immunogenic, inducing broad, polyfunctional and long-lasting CD4^+^ and CD8^+^ T cell responses to HIV-1 antigens, with preference for effector memory T cells; and also induced antibody responses to Env in 95% of volunteers [19], [20].

However, more efficient poxvirus MVA-B vectors that enhance the magnitude, breath, polyfunctionality and durability of the immune responses to HIV-1 antigens are desirable. The MVA vector still contains viral genes with immunomodulatory functions that may suppress host immunity, and in particular, it encodes inhibitors of the interferon (IFN) signaling pathway (one of the main routes involved in the host innate immunity developed to fight viral infections and prevent the pathogenesis of virus-induced diseases), which blocks the induction of host defense mechanisms [21]. Therefore, deletion in the vector backbone of MVA of these immunomodulatory VACV genes is a possible attractive strategy for improving the safety and immunogenicity properties of recombinants based on the MVA strain.

Recently, we have reported that improved NYVAC-C recombinants expressing the HIV-1 Env and Gag-Pol-Nef antigens from clade C, that contains deletions in VACV virus genes *B8R* and *B19R* (which act blocking the binding of IFN to its receptor), were able to enhance HIV-1-specific immune responses [22], [23], [24]. Since *B8R* and *B19R* genes are non functional or deleted in the MVA genome [25], [26], we proposed to follow a similar strategy to that used with NYVAC-C, but deleting VACV virus genes, present in MVA, acting intracellularly to block the IFN signaling pathway, such as VACV virus genes *C6L* and *K7R*.

The VACV *C6L* gene encodes for a non-essential protein expressed early during infection [15], [27], that is a member of the VACV Bcl-2 family [28]. *C6L* encodes for a protein which act inhibiting the expression of IFN-β following stimulation of cells with multiple Toll-like receptor- and RIG-I-like receptor- ligands by preventing the translocation of IFN regulatory factor (IRF)-3 into the nucleus [27]. C6 protein interacts with TANK, NAP1 and SINTBAD, scaffold proteins that are constitutively associated with TBK1 and IKKε [27]. *C6L* is absent in NYVAC, but present in MVA. Deletion of *C6L* in the vector backbone of the HIV/AIDS vaccine candidate MVA-B, enhanced HIV-1–specific cellular and humoral immune responses following a DNA prime/MVA boost immunization protocol in mice, and induced an up-regulation of the expression of IFN-β and IFN-α/β-inducible genes in human macrophages and monocyte-derived dendritic cells (moDCs) [15]. Furthermore, a VACV Western Reserve (WR) strain with a deletion in *C6L* showed enhanced VACV-specific cytotoxic T cell responses and resulted in a more efficacious vaccine that provided better protection against challenge with VACV [29].

On the other hand, VACV *K7R* gene also belongs to the VACV Bcl-2 family [28], and encodes for the K7 protein, whose crystal structure has been solved [30]. K7 inhibits innate immune signaling pathways [31], acting as an intracellular inhibitor of both NF-κB and IRF3 activation [32]. K7 inhibits TLR-induced NF-κB activation by interaction with IRAK2 and TRAF6 [32]. Moreover, K7 binds to the cellular DEAD-box RNA helicase DDX3 [31], which forms part of a complex containing TBK1 and IKKε, that activates IRF3, thus inhibiting IRF3/7 phosphorylation and in consequence induction of the IFN-β promoter and IFN-β gene transcription [32].

To define the role of viral genes that act on the IFN-regulatory factor IRF-3, thus preventing induction of IFN-β, here, we have generated MVA-B recombinants with deletions in VACV genes blocking the IFN signaling pathway at the intracellular level (such as *C6L* and *K7R*). We have examined the innate immune signaling profile elicited in human THP-1 cells and monocyte-derived dendritic cells (moDCs), and evaluated whether these viral inhibitors of the IFN system impact the capacity of the MVA-B vector to activate HIV-1-specific adaptive and memory immune responses in a mouse model. The results obtained revealed that a double deletion of viral factors (C6 and K7) targeting the IFN pathway intracellularly is a useful approach in the design of improved poxvirus-based vaccines.

## Results

### Generation and *in vitro* characterization of MVA-B deletion mutants

**Figure 1:**
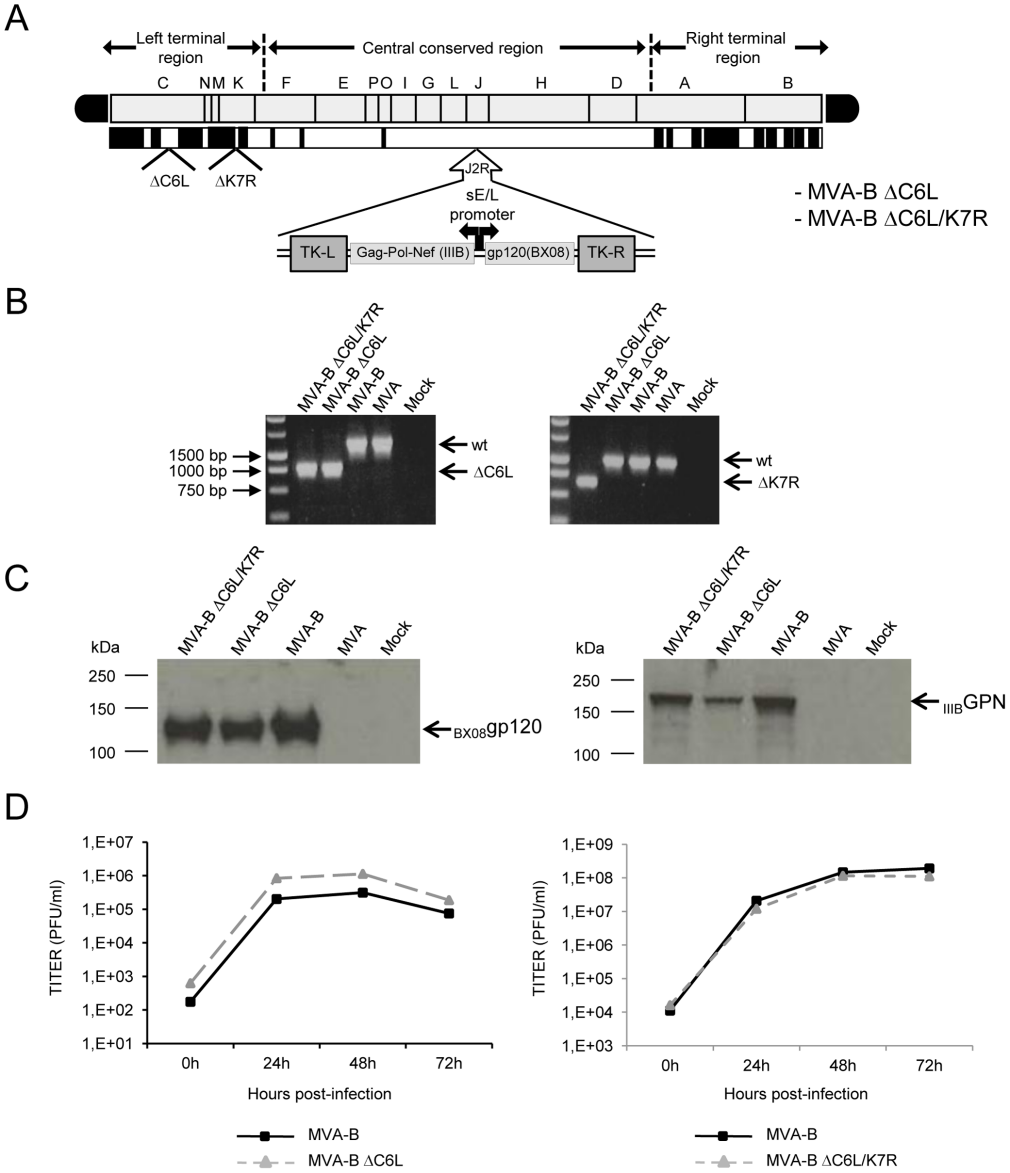
*In vitro* characterization of MVA-B deletion mutants. (A) Scheme of MVA-B deletion mutant's genome map, adapted from [25] and [69]. The different regions are indicated by capital letters. The right and left terminal regions are shown. Below the map, the deleted or fragmented genes are depicted as black boxes. The deleted *C6L* and *K7R* genes are indicated. The HIV-1 Gag-Pol-Nef (from isolate IIIB) and gp120 (from isolate BX08) clade B sequences driven by the synthetic early/late (sE/L) virus promoter inserted within the TK viral locus (J2R) are indicated (adapted from [16]). (B) PCR analysis of *C6L* and *K7R* loci. Viral DNA was extracted from DF-1 cells mock-infected or infected at 1 PFU/cell with MVA-WT, MVA-B, MVA-B ΔC6L or MVA-B ΔC6L/K7R. Primers spanning *C6L- or K7R-*flanking regions were used for PCR analysis of the *C6L* or *K7R* loci, respectively. The DNA products corresponding to the parental virus or to the deletion are indicated by an arrow on the right. Molecular size marker (1 Kb ladder) with the corresponding sizes (base pairs) is indicated on the left. Lane Mock, cells not infected. (C) Expression of HIV-1 _BX08_gp120 and _IIIB_GPN proteins. DF-1 cells were mock-infected or infected at 1 PFU/cell with MVA-WT, MVA-B, MVA-B ΔC6L or MVA-B ΔC6L/K7R. At 24 hours post-infection, cells were lysed in Laemmli buffer, fractionated by 8% SDS-PAGE and analyzed by Western-blot using rabbit polyclonal anti-gp120 antibody or polyclonal anti-gag p24 serum. Arrows on the right indicate the position of HIV-1 _BX08_gp120 and _IIIB_GPN proteins. (D) Viral growth kinetics in DF-1 cells. DF-1 cells were infected at 0.01 PFU/cell with MVA-B, MVA-B ΔC6L or MVA-B ΔC6L/K7R. At different times post-infection (0, 24, 48, and 72 hours), cells were harvested and virus titers in cell lysates were determined by plaque immunostaining assay with anti-WR antibodies. The mean of two independent experiments is shown.

To determine whether immunomodulatory VACV genes blocking the IFN signaling pathway at the intracellular level (such as *C6L* and *K7R*) could affect the immunogenicity profile against HIV-1, we have constructed a single and a double MVA-B deletion mutant lacking VACV genes C6L and/or K7R (viruses termed MVA-B ΔC6L and MVA-B ΔC6L/K7R; see Materials and Methods) from the HIV/AIDS vaccine candidate MVA-B (expressing HIV-1 Env, Gag, Pol and Nef antigens from clade B) [16]. A diagram of these MVA-B deletion mutants is shown in Figure 1A. PCR using primers for the *C6L* and *K7R* loci confirmed the deletion of *C6L* from MVA-B ΔC6L, and *C6L* and *K7R* from MVA-B ΔC6L/K7R (Figure 1B). Moreover, the correct generation and purity of each deletion mutant were also confirmed by DNA sequencing (data not shown). Additionally, analysis by Western blot demonstrated that the MVA-B deletion mutants expressed correctly the HIV-1 antigens _BX08_gp120 and _IIIB_GPN, with the same size as their parental virus, MVA-B (Figure 1C). Furthermore, analysis by immunostaining showed that all virus plaques had immunoreactivities to both anti-WR and anti-gp120 antibodies similar to the parental MVA-B (data not shown), demonstrating the stability of the viruses.

### C6 and K7 are non-essential in cell culture

The mere isolation of the different MVA-B deletion mutants confirmed that C6 and K7 proteins are not essential for MVA replication. However, to further characterize whether single or double deletions of *C6L* and/or *K7R* VACV genes affected virus replication in cell cultures, we compared the growth kinetics in DF-1 cells of the different MVA-B deletion mutants with their parental virus, MVA-B. The results showed that the kinetics of growth was similar between parental MVA-B and each MVA-B deletion mutant (Figure 1D). Therefore, when deleted individually or in double, C6 and K7 VACV proteins are not required for virus replication in cultured cells.

### MVA-B ΔC6L/K7R up-regulate IFN-β, TNF-α and MIP-1α expression in human macrophages and dendritic cells

**Figure 2:**
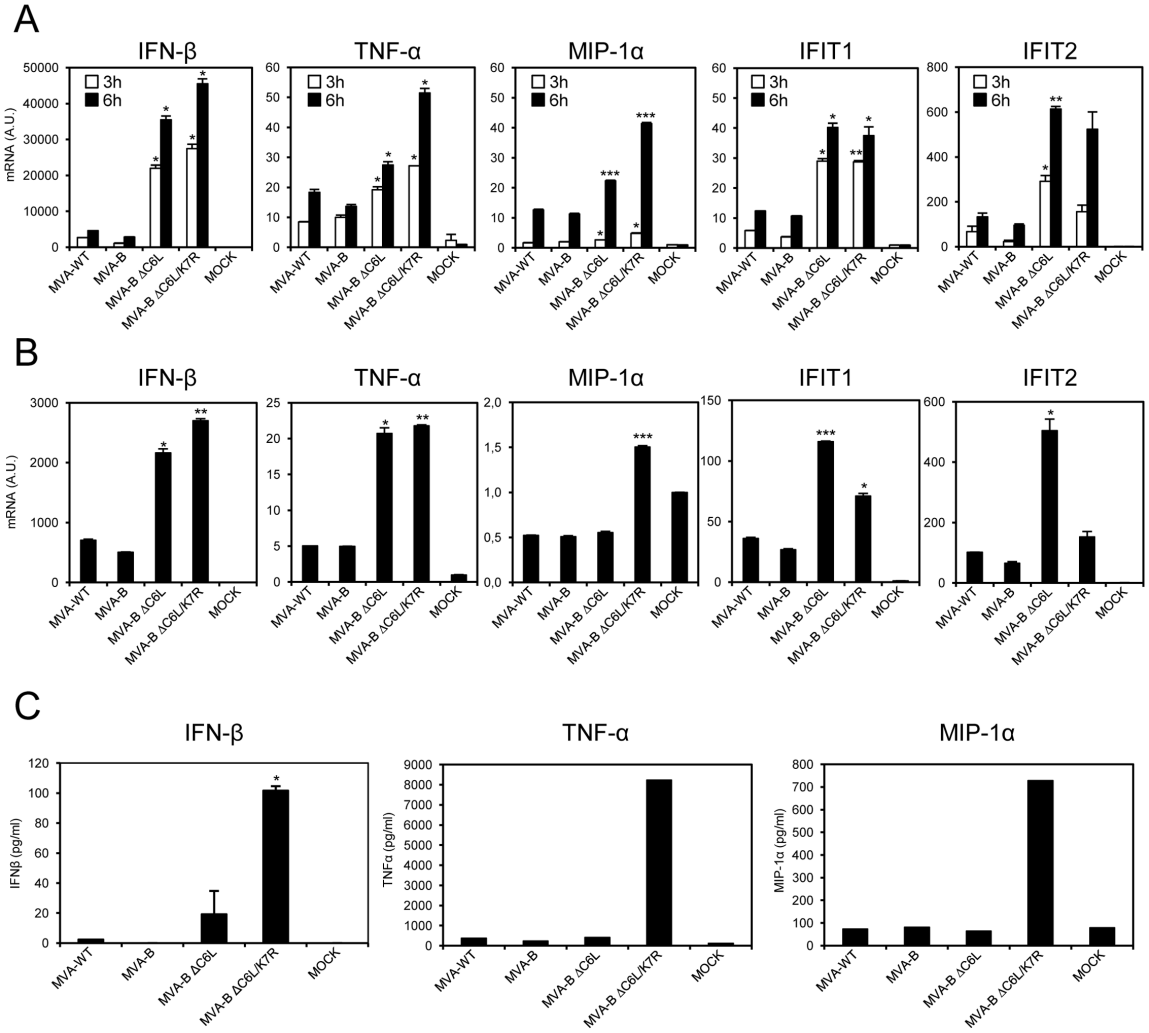
MVA-B deletion mutants induces the production of IFN-β, type I IFN inducible genes, TNF-α and MIP-1α in macrophages and dendritic cells. Human THP-1 macrophages (A) and moDCs (B, C) were mock-infected or infected with MVA-WT, MVA-B, MVA-B ΔC6L or MVA-B ΔC6L/K7R (5 PFU/cell in A, and 1 PFU/cell in B and C). At different time post-infection (3 h and 6 h in A, 6 h in B), RNA was extracted and the mRNA levels of IFN-β, TNF-α, MIP-1α, type I IFN inducible genes (IFIT1 and IFIT2), and HPRT were analyzed by RT-PCR. Results were expressed as the ratio of gene to HPRT mRNA levels. A.U: arbitrary units. *p* values indicate significantly higher responses compared to DNA-B/MVA-B immunization group. * *p*<0.05, ** *p*<0.005, *** *p*<0.001. Data are means ± SD of duplicate samples and are representative of three independent experiments. (C) Human moDCs were mock-infected or infected with 1 PFU/cell of MVA-WT, MVA-B, MVA-B ΔC6L or MVA-B ΔC6L/K7R. Six hours later, cell-free supernatants were collected to quantify the concentration of IFN-β by ELISA and the concentration of TNF-α and MIP-1α by Luminex. Data are means ± SD of duplicates and are representative of three independent experiments.

To determine whether C6 and K7 impair the response of innate immune cells to MVA-B, we examined by real time PCR the expression of IFN-β, TNF-α, MIP-1α and IFN-β-induced genes (IFIT1, IFIT2) by human THP-1 macrophages infected for 3 and 6 hours with 5 PFU/cell of MVA-WT, MVA-B, MVA-B ΔC6L and MVA-B ΔC6L/K7R (Figure 2A). Compared to MVA-WT and MVA-B, the MVA-B deletion mutants markedly up-regulated the expression of IFN-β, TNFα, MIP-1α, as well as IFN-β-dependent genes (IFIT1 and IFIT2) in THP-1 cells. Interestingly, MVA-B ΔC6L/K7R was the higher inducer of IFN-β, TNFα and MIP-1α (Figure 2A).

To verify that C6 and K7 impaired IFN-β, TNF-α, MIP-1α and IFN-β-dependent gene expression in innate immune cells, we infected human moDCs with 1 PFU/cell of MVA-WT, MVA-B, MVA-B ΔC6L and MVA-B ΔC6L/K7R and measured IFN-β, TNF-α, MIP-1α, IFIT1 and IFIT2 mRNA levels 6 h post-infection (Figure 2B). Similarly to the results obtained with human THP-1 cells, MVA-B deletion mutants strongly increased in moDCs IFN-β, TNF-α and MIP-1α expression, compared to MVA-WT and MVA-B. Again, MVA-B ΔC6L/K7R was the highest inducer of IFN-β, TNFα and MIP-1α (Figure 2B).

In addition we measured protein levels of IFN-β, TNFα and MIP-1α levels in the supernatant from moDCs mock-infected or infected for 6 h with 1 PFU/cell of MVA-WT, MVA-B, MVA-B ΔC6L and MVA-B ΔC6L/K7R (Figure 2C). The results showed that MVA-B ΔC6L/K7R significantly enhanced the release by moDCs of higher levels of IFN-β, TNFα and MIP-1α than parental or single C6 deletion mutant.

Thus, double deletion of C6 and K7 in the MVA-B genome promotes strong innate immune expression of IFN-β, TNFα and MIP-1α production in human macrophages and moDCs.

### MVA-B ΔC6L/K7R enhances the magnitude and polyfunctionality of HIV-1-specific T cell adaptive immune responses

Given the immunomodulatory roles of C6 and K7, we next defined immune responses induced by the single or double deletions of *C6L* and/or *K7R* VACV genes in BALB/c mice inoculated in prime/boost immunization protocols, using a DNA prime (100 µg of DNA-B, i.m.)/MVA boost (1 x 10^7^ PFU, i.p.) (see Materials and Methods). Animals primed with sham DNA (DNA-φ) and boosted with non-recombinant MVA-WT were used as a control group. Adaptive HIV-1-specific T cell immune responses elicited by the immunization groups were measured 10 days after the boost by polychromatic intracellular cytokine staining (ICS) assay, after the stimulation of splenocytes with pools of peptides (Env-pool, Gag-pool and GPN-pool) that spanned the HIV-1 Env, Gag, Pol and Nef regions from clade B included in the immunogens.

**Figure 3:**
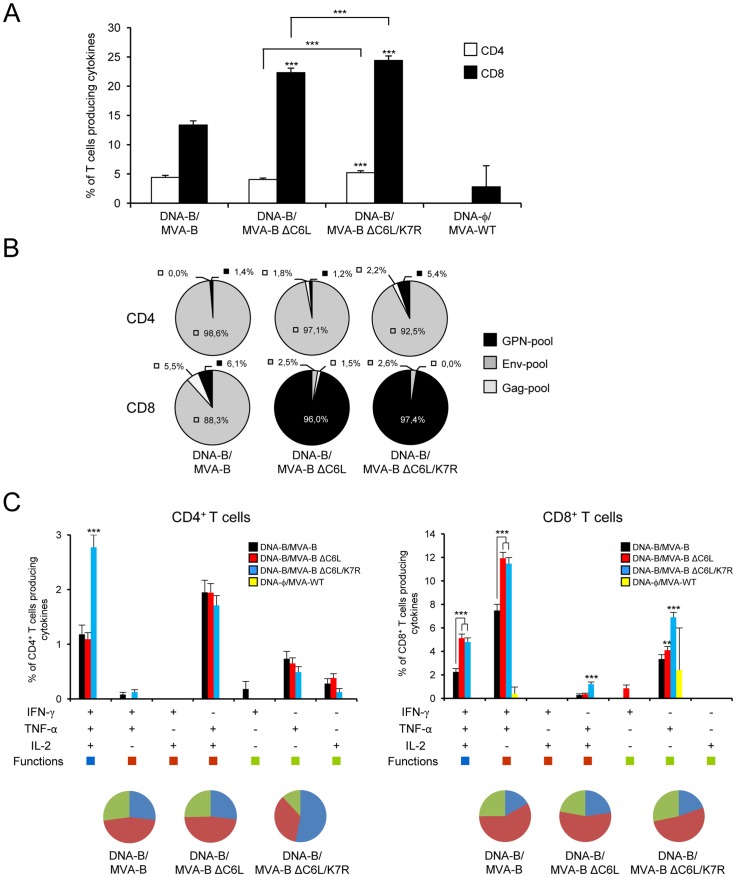
Immunization with MVA-B deletion mutants enhances the magnitude and polyfunctionality of HIV-1-specific CD4^+^ and CD8^+^ T cell adaptive immune responses. Splenocytes were collected from mice (n = 4 per group) immunized with DNA-φ/MVA-WT, DNA-B/MVA-B, DNA-B/MVA-B ΔC6L or DNA-B/MVA-B ΔC6L/K7R 10 days after the last immunization. Then, HIV-1-specific CD4^+^ and CD8^+^ T cell adaptive immune responses elicited by the different immunization groups were measured by ICS assay following stimulation of splenocytes with different HIV-1 peptide pools (Env-pool, Gag-pool and GPN-pool). Values from unstimulated controls were subtracted in all cases. *p* values indicate significantly higher responses compared to DNA-B/MVA-B immunization group, and also between MVA-B deletion mutants. Data are from one experiment representative of three experiments. (A) Magnitude of HIV-1-specific CD4^+^ and CD8^+^ T cells. The values represent the sum of the percentages of T cells secreting IFN-γ and/or TNF-α and/or IL-2 against Env+Gag+GPN peptide pools. *** *p*<0.001. (B) Percentage of Env, Gag and GPN HIV-1-specific CD4^+^ and CD8^+^ T cell adaptive immune responses. Frequencies were calculated by reporting the number of T cells producing IFN-γ and/or TNF-α and/or IL-2 to the total number of CD4^+^ and CD8^+^ T cells in the different immunization groups. (C) Polyfunctionality of HIV-1-specific CD4^+^ and CD8^+^ T cells. Functional profiles of HIV-1-specific CD4^+^ (left panel) and CD8^+^ (right panel) T cell adaptive immune responses in the different immunization groups. Responses are grouped and color-coded on the basis of the number of functions. All the possible combinations of the responses are shown on the X axis, while the percentages of T cells secreting IFN-γ and/or TNF-α and/or IL-2 against Env+Gag+GPN peptide pools are shown on the Y axis. The pie charts summarize the data. Each slice corresponds to the proportion of HIV-1-specific CD4^+^ or CD8^+^ T cells producing one, two or three cytokines (IFN-γ and/or TNF-α and/or IL-2) within the total HIV-1-specific CD4^+^ or CD8^+^ T cells. * *p*<0.05, ** *p*<0.005, *** *p*<0.001.

The magnitude of HIV-1-specific CD4^+^ and CD8^+^ T cell adaptive immune responses, determined as the sum of the individual responses producing IFN-γ, TNF-α and/or IL-2 obtained for Env, Gag and GPN peptide pools, was significantly higher in the immunization groups with the recombinant vectors than that elicited by the control group, non recombinant DNA/MVA-WT (p<0.001) (Figure 3A). The overall HIV-1-specific immune response was mainly mediated by CD8^+^ T cells (75–84%) in the immunization groups (Figure 3A). However, immunization with DNA-B/MVA-B ΔC6L and DNA-B/MVA-B ΔC6L/K7R induced a significantly higher magnitude of HIV-1-specific CD8^+^ T cell responses than DNA-B/MVA-B (p<0.001); and DNA-B/MVA-B ΔC6L/K7R induced a significantly higher magnitude of HIV-1-specific CD4^+^ T cell responses than DNA-B/MVA-B (p<0.001) (Figure 3A).

The pattern of the HIV-1-specific T cell adaptive immune response is represented in Figure 3B. The CD4^+^ T cell responses were mainly directed against the Env-pool (>92%) in all the immunization groups, with no differences between the groups boosted with MVA-B deletion mutants and the group boosted with the parental MVA-B. However, the pattern of the CD8^+^ T cell responses was clearly different between the parental MVA-B and the MVA-B deletion mutants: DNA-B/MVA-B induced preferentially Env-specific CD8^+^ T cell responses (>88%), while the DNA-B/MVA-B deletion mutants induced a higher percentage of GPN-specific CD8^+^ T cell responses (>96%). Similar findings were observed in three independent experiments.

The quality of the HIV-1-specific T cell adaptive immune response was characterized by the production of IFN-γ, TNF-α and/or IL-2 cytokines from HIV-1-specific CD4^+^ and CD8^+^ T cells (Figure 3C). Seven distinct HIV-1-specific CD4^+^ and CD8^+^ T cell populations were identified. HIV-1-specific CD4^+^ T cell responses were highly polyfunctional in all the immunization groups (>73% CD4^+^ T cells exhibiting two or three functions), with DNA-B/MVA-B ΔC6L/K7R having the highest polyfunctionality (>88% CD4^+^ T cells producing two or three cytokines). CD4^+^ T cells producing IFN-γ + TNF-α + IL-2, TNF-α + IL-2 or only TNF-α were the most induced populations elicited by the parental MVA-B and the MVA-B deletion mutants, with DNA-B/MVA-B ΔC6L/K7R inducing a significant increase in the percentage of triple positive CD4^+^ T cells secreting IFN-γ + TNF-α + IL-2, compared to MVA-B (p<0.001) (Figure 3C, left panel). HIV-1-specific CD8^+^ T cell responses were also high and of similar polyfunctionality profile in all the immunization groups (>72% CD8^+^ T cells exhibiting two or three functions). CD8^+^ T cells producing IFN-γ + TNF-α + IL-2, IFN-γ + TNF-α or only TNF-α were the most abundant populations elicited by the parental MVA-B and the MVA-B deletion mutants. However, DNA-B/MVA-B ΔC6L and DNA-B/MVA-B ΔC6L/K7R induced a significantly higher increase in the percentage of each of these populations, compared to MVA-B (p<0.001) (Figure 3C, right panel).

Altogether, these findings revealed that single or double deletion of *C6L* and/or *K7R* VACV genes enhanced the magnitude and quality of HIV-1-specific T cell adaptive immune responses, being MVA-B ΔC6L/K7R the most immunogenic.

### T cell memory immune responses are improved by MVA-B ΔC6L/K7R

HIV-1-specific T cell memory immune responses elicited by the immunization groups were carried out 52 days after the boost by ICS assay, similarly to the protocol followed in the adaptive phase.

**Figure 4:**
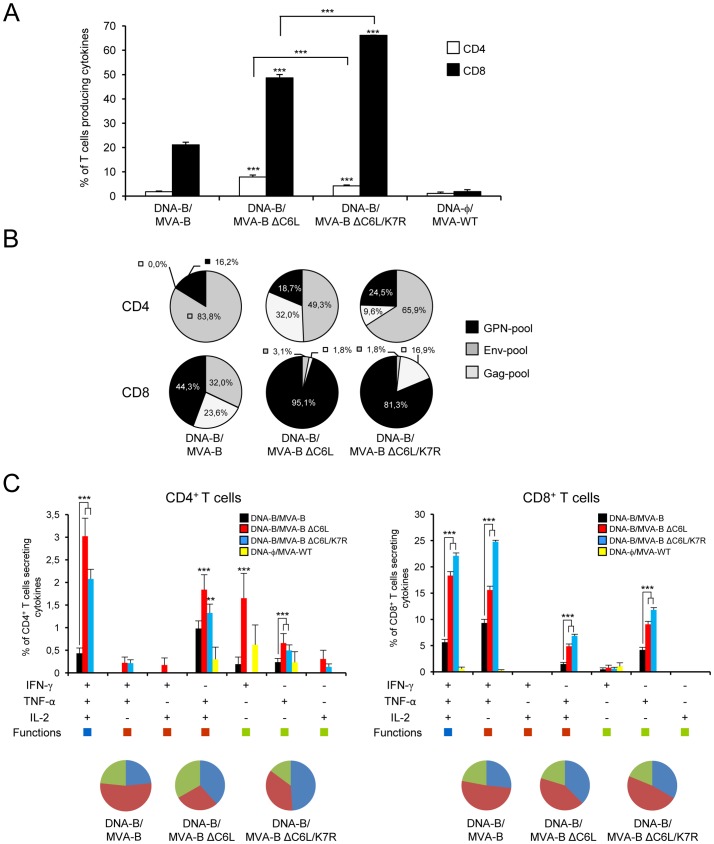
Immunization with MVA-B deletion mutants enhances the magnitude and polyfunctionality of HIV-1-specific CD4^+^ and CD8^+^ T cell memory immune responses. Splenocytes were collected from mice (n = 4 per group) immunized with DNA-φ/MVA-WT, DNA-B/MVA-B, DNA-B/MVA-B ΔC6L or DNA-B/MVA-B ΔC6L/K7R 52 days after the last immunization and HIV-1-specific CD4^+^ and CD8^+^ T cell memory immune responses were analyzed by flow cytometry as described in Figure 3. Values from unstimulated controls were subtracted in all cases. *p* values indicate significantly higher responses compared to DNA-B/MVA-B immunization group, and also between MVA-B deletion mutants. Data are from one experiment representative of three experiments. (A) Magnitude of HIV-1-specific CD4^+^ and CD8^+^ T cells. The values represent the sum of the percentages of T cells secreting IFN-γ and/or TNF-α and/or IL-2 against Env+Gag+GPN peptide pools. *** *p*<0.001. (B) Percentage of Env, Gag and GPN HIV-1-specific CD4^+^ and CD8^+^ T cell memory immune responses. Frequencies were calculated by reporting the number of T cells producing IFN-γ and/or TNF-α and/or IL-2 to the total number of CD4^+^ and CD8^+^ T cells in the different immunization groups. (C) Polyfunctionality of HIV-1-specific CD4^+^ and CD8^+^ T cells. Functional profile of HIV-1-specific CD4^+^ (left panel) and CD8^+^ (right panel) T cell memory immune responses in the different immunization groups. Responses are grouped and color-coded on the basis of the number of functions. All the possible combinations of the responses are shown on the X axis. The percentages of T cells secreting IFN-γ and/or TNF-α and/or IL-2 against Env+Gag+GPN peptide pools are shown on the Y axis. The pie charts summarize the data. Each slice corresponds to the proportion of HIV-1-specific CD4^+^ or CD8^+^ T cells producing one, two or three cytokines (IFN-γ and/or TNF-α and/or IL-2) within the total HIV-1-specific CD4^+^ or CD8^+^ T cells. ** *p*<0.005, *** *p*<0.001.

The magnitude of HIV-1-specific CD4^+^ and CD8^+^ T cell memory immune responses, determined as the sum of the individual responses producing IFN-γ, TNF-α and/or IL-2 obtained for Env, Gag and GPN peptide pools, was significantly higher in the immunization groups with recombinant vectors than that elicited by the control group, DNA-φ/MVA-WT (p<0.001) (Figure 4A). Similarly to the results obtained in the adaptive phase, the overall HIV-1-specific T cell memory immune response was mainly mediated by CD8^+^ T cells (86–94%) in all the immunization groups (Figure 4A). However, immunization with DNA-B/MVA-B ΔC6L and DNA-B/MVA-B ΔC6L/K7R again induced a significantly higher magnitude of HIV-1-specific CD8^+^ T cell responses than DNA-B/MVA-B (p<0.001); and the MVA-B deletion mutants induced a significantly higher magnitude of HIV-1-specific CD4^+^ T cell responses than DNA-B/MVA-B (p<0.001) (Figure 4A).

The pattern of HIV-1-specific CD4^+^ T cell memory immune responses was similar in all the immunization groups, with responses mainly directed against the Env-pool (49–83%) (Figure 4B), in the same way to the results obtained in the adaptive phase. However, the pattern of the CD8^+^ T cell memory responses was clearly different between the parental MVA-B and the MVA-B deletion mutants: DNA-B/MVA-B induced a more broadly distributed response between Env-, Gag- and GPN-specific CD8^+^ T cell memory responses, while the DNA-B/MVA-B deletion mutants induced a higher percentage of GPN-specific CD8^+^ T cell memory responses (>81%) (Figure 4B), similarly to the pattern obtained in the adaptive phase. Similar findings were observed in three independent experiments.

The quality of the HIV-1-specific T cell memory immune responses was characterized analyzing the production of IFN-γ, TNF-α and/or IL-2 cytokines by HIV-1-specific CD4^+^ and CD8^+^ T cells (Figure 4C), similarly to the protocol followed in the adaptive phase. HIV-1-specific CD4^+^ T cell memory responses were highly polyfunctional in all the immunization groups (>67% CD4^+^ T cells exhibiting two or three functions), with DNA-B/MVA-B ΔC6L/K7R been the highest polyfunctional (>85% CD4^+^ T cells producing two or three cytokines), similarly to the results obtained in the adaptive phase. Again, CD4^+^ T cells producing IFN-γ + TNF-α + IL-2 or TNF-α + IL-2 were the most induced populations elicited by the parental MVA-B and the MVA-B deletion mutants, with the MVA-B deletion mutants inducing a significantly higher increase in the percentage of these CD4^+^ T cells populations, compared to MVA-B (p<0.001) (Figure 4C, left panel). HIV-1-specific CD8^+^ T cell memory responses were also highly polyfunctional in all the immunization groups (>78% CD8^+^ T cells exhibiting two or three functions), similarly to the results obtained in the adaptive phase. Again, CD8^+^ T cells producing IFN-γ + TNF-α + IL-2, IFN-γ + TNF-α or only TNF-α were the most frequent induced populations elicited by the parental MVA-B and the MVA-B deletion mutants. However, DNA-B/MVA-B ΔC6L and DNA-B/MVA-B ΔC6L/K7R induced a significantly higher increase in the percentage of each of these populations, compared to MVA-B (p<0.001) (Figure 4C, right panel).

**Figure 5:**
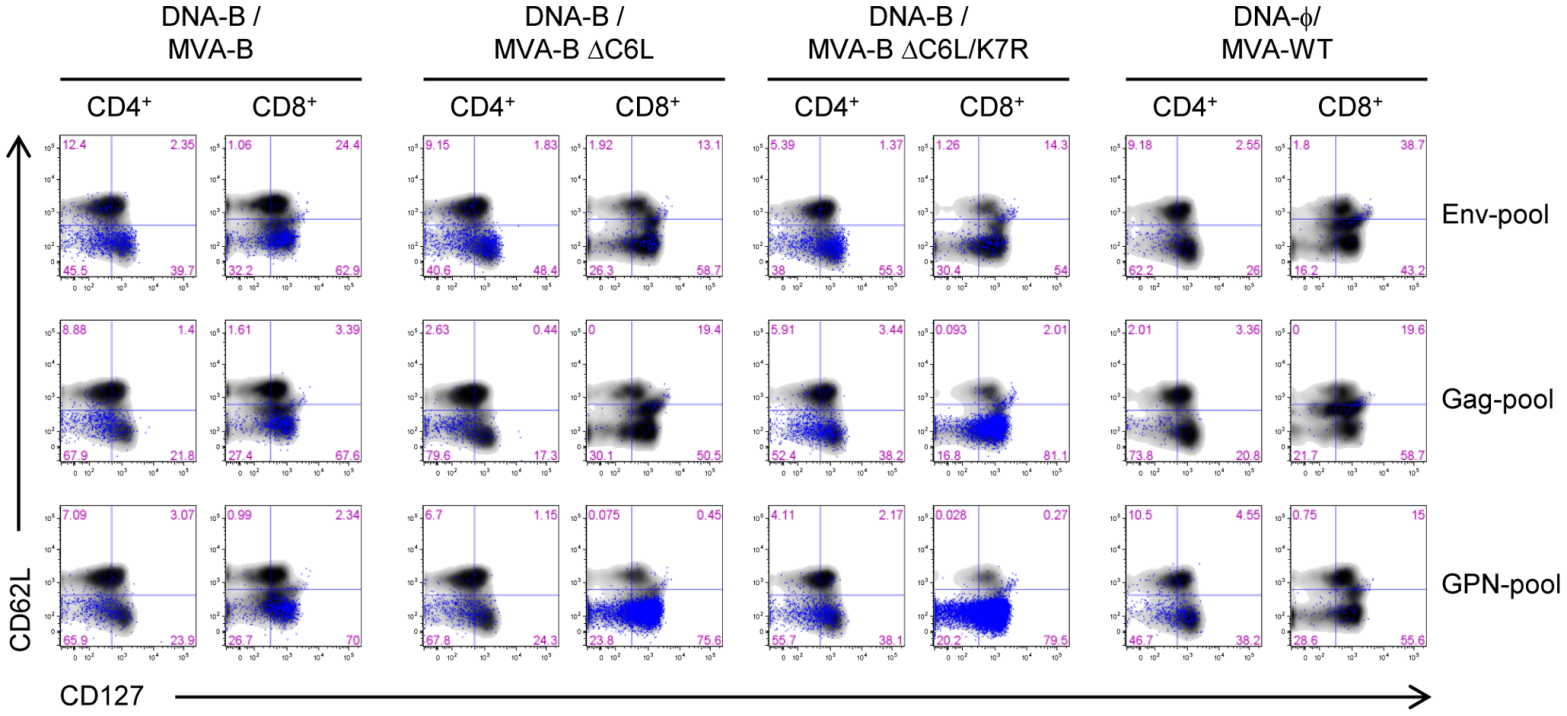
Phenotypic profile of memory HIV-1-specific CD4^+^ and CD8^+^ T cells. FACS plots of HIV-1 Env-, Gag- and GPN-specific CD4^+^ and CD8^+^ T memory cells. CD127 and CD62L expression was used to identify central memory (Tcm: CD127^+^/CD62L^+^), effector memory (Tem CD127^+^/CD62L^−^) and effector (Te: CD127^−^/CD62L^−^) sub-populations. The T cell memory sub-populations are depicted as density plots. Blue dots represent T cells producing IFN-γ and/or TNF-α and/or IL-2. Data are from one experiment representative of three experiments.

Additionally, we also determined the phenotype of the HIV-1-specific CD4^+^ and CD8^+^ T memory cells by measuring the expression of CD127 and CD62L surface markers, which allow to define the different memory sub-populations: central memory (T_CM_: CD127^+^/CD62L^+^), effector memory (T_EM_: CD127^+^/CD62L^−^) and effector (T_E_: CD127^−^/CD62L^−^) T cells. As shown in Figure 5, in all the immunization groups, HIV-1-specific CD4^+^ and CD8^+^ T memory cells were mainly of the T_EM_ phenotype. However, MVA-B deletion mutants induced an increase in the percentage of some HIV-1-specific CD4^+^ and CD8^+^ T_EM_ cells. For example, in DNA-B/MVA-B 39% of the Env-specific CD4^+^ T cells were of the T_EM_ phenotype, whereas immunization with MVA-B deletion mutants increased this population up to 55%, in the case of DNA-B/MVA-B ΔC6L/K7R. Furthermore, in DNA-B/MVA-B 70% of the GPN-specific CD8^+^ T cells were of the T_EM_ phenotype, whereas immunization with MVA-B deletion mutants increased this population up to 80%, in the case of DNA-B/MVA-B ΔC6L/K7R.

Overall, these results revealed that single or double deletion of *C6L* and/or *K7R* VACV genes enhanced the magnitude and quality of HIV-1-specific T cell memory immune responses, with the highest responses induced by DNA-B/MVA-B ΔC6L/K7R.

### MVA-B ΔC6L/K7R slightly enhances levels of antibodies against HIV-1 gp120

**Figure 6:**
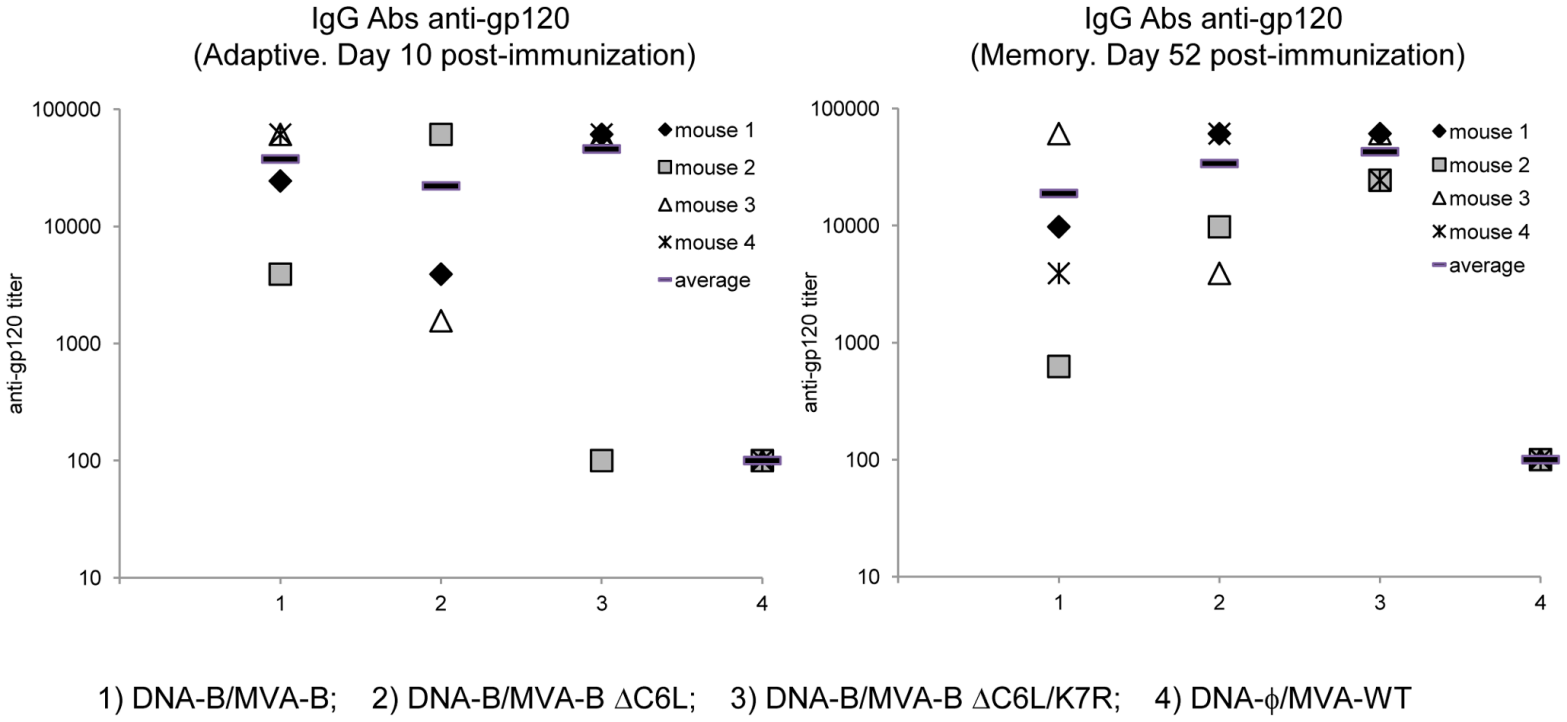
Humoral immune responses elicited by MVA-B and MVA-B deletion mutants against HIV-1 gp120 protein. Serum was collected from individual mouse immunized with DNA-B/MVA-B, DNA-B/MVA-B ΔC6L or DNA-B/MVA-B ΔC6L/K7R or DNA-φ/MVA-WT (n = 4), 10 days (left panel) or 52 days (right panel) after the last immunization. Levels of gp120-specific IgG binding antibodies were measured by ELISA. Titers represent the last dilution of the serum that signals 3-fold higher than signals obtained with the serum of naïve mice. The horizontal bar represents the mean antibodies titer for each group. Each dot represents data obtained from one mouse. Data are from one experiment representative of three experiments.

Since cells infected with MVA-B release monomeric gp120 [16], we evaluated whether immunization with MVA-B or MVA-B deletion mutants stimulated the production of antibodies against HIV-1 Env in the serum obtained from individual mouse 10 and 52 days post-boost. Env-specific IgG titers against the purified gp120 protein from the HIV-1 isolate MN (clade B) were quantified by ELISA (Figure 6). Although not significant, in the groups boosted with the MVA-B deletion mutants, the levels of anti-gp120 antibodies were slightly enhanced compared to those obtained in animals immunized with the parental MVA-B, mainly in the memory phase (day 52), with MVA-B ΔC6L/K7R inducing the higher levels of antibodies. The exact values of the mean titer of anti-gp120 antibodies are: 37.598 for DNA-B/MVA-B and 45.801 for DNA-B/MVA-B ΔC6L/K7R, in the adaptive phase (increase in 1.21-fold); and 18.833 for DNA-B/MVA-B and 42.725 for DNA-B/MVA-B ΔC6L/K7R, in the memory phase (increase in 2.26-fold). These results indicate that single and/or double deletion of *C6L* and/or *K7R* VACV genes produces in mice a slightly effect on the humoral immune response against HIV-1 Env.

## Discussion

Vaccinia virus recombinants with enhanced ability to induce immune responses to clinically relevant antigens delivered from the virus vector is an expanding field in vaccine design, raising the interest of poxvirus vectors as vaccine candidates against multiple diseases. In fact, different poxvirus vectors, like canarypox, NYVAC, MVA and fowlpox, are in human clinical trials, with the aim to understand their behavior as immunogens and potency against different pathogens and tumors [1], [2], [4], [33]. Among them, we have developed an HIV/AIDS vaccine candidate using an MVA vector which contains Env, Gag, Pol and Nef antigens of HIV-1 from clade B (termed MVA-B), that has been well characterized *in vitro* [34], [35], in mouse models [14], [15], [16], [17], non-human primates [18], and in a phase I clinical trial in humans [19], [20], showing that MVA-B is a promising HIV/AIDS vaccine candidate, as defined by high immunogenicity, ability to induce broad, polyfunctional and long-lasting CD4^+^ and CD8^+^ T cell responses to HIV-1 antigens. Moreover, the results of the RV144 phase III clinical trial for HIV/AIDS highlighted that improved poxvirus vectors should be sought, as the protection observed in the Thailand trial against HIV-1 was modest (31%) [3].

Among the different approaches developed to enhance the immune response of the poxvirus vectors, one promising strategy is the removal of VACV genes that antagonize with the immune system, as the virus genome still contains several genes that interfere with the host immune responses [21], [36]. These viral immune modulators can block immune pathways at different levels. One important pathway that is antagonized by VACV genes is the IFN system, where VACV immunomodulators can act either at the extracellular level by sequestering soluble IFN and thus blocking ligand-receptor interaction, or act at the intracellular level by blocking the NF-κB and IRF signaling pathways [21], [36]. We have previously shown that VACV genes *B8R* and *B19R*, known to antagonize type I and type II IFN ligand-receptor interaction [37], [38], when deleted in the NYVAC vector develop in mice an enhanced immune response to HIV-1 antigens expressed from the recombinant virus [22], [23], [24]. To extend those studies, here we have investigated the consequences of deleting in the MVA strain two other VACV genes that act on the IFN intracellular signaling pathway (*C6L* and *K7R*), and have evaluated the capacity of mutant vectors to trigger HIV-1 specific immune responses. The relevance of these two viral genes is that both act differently but on similar IFN signaling steps, namely, inhibiting the IRF3/IFN-β regulatory pathways. This has been done in human macrophages and moDC cells infected with recombinant virus and in a mouse model (BALB/c) following DNA prime/pox boost protocols of immunization [14], [15]. We have compared the type of immune responses (innate, adaptive and memory) triggered by three vectors: the parental MVA-B, a single deletion mutant (MVA-B ΔC6L) and a double deletion mutant (MVA-B ΔC6L/K7R). Our findings showed that MVA-B ΔC6L/K7R is the most immunogenic vector promoting strong innate immune expression of IFN-β, TNFα and MIP-1α production in human macrophages and moDCs. Furthermore, in DNA prime/pox boost immunization protocols, MVA-B ΔC6L/K7R significantly enhanced the magnitude and quality of HIV-1-specific CD4^+^ and CD8^+^ T cell immune responses during the adaptive and memory phases. MVA-B ΔC6L and MVA-B ΔC6L/K7R triggered CD4^+^ and CD8^+^ T cells of a polyfunctional phenotype with triple IFN-γ + TNF-α + IL-2 (in the case of CD4^+^ and CD8^+^ T cells), double TNF-α + IL-2 (in the case of CD4^+^ T cells) or double IFN-γ + TNF-α (in the case of CD8^+^ T cells) cytokine T cell producers. In all the immunization groups, the HIV-1 specific T cell responses were mainly mediated by CD8^+^ T cells, similarly to previous results with MVA or NYVAC vectors containing deletions in VACV genes [14], [15], [22], [39]. This preferential induction of HIV-1 specific CD8^+^ T cells by the MVA-B and MVA-B deletion mutants might be due to the inherent innate immune-modulatory effects of the MVA vector on target cells. In fact, we have previously reported that co-culture of MVA-B infected monocyte-derived DCs with autologous T lymphocytes from HIV-infected individuals induced a highly functional HIV-1 specific CD8^+^ T cell response, with increased expansion of antigen-specific CD8^+^ T cells [34]. Furthermore, expression of HIV-1 proteins from moDCs infected with MVA-B induced the expression of type I IFNs, chemokines and pro-inflammatory cytokines, that might act as regulators of immune responses to HIV-1 antigens [35], promoting a main increase in HIV-1 specific CD8^+^ T cells. Deletion of *C6L* and *K7R* increased type I IFN and cytokine/chemokine production, which could lead to a better recruitment of immature DCs and lymphocytes, generating a suitable environment for the cross-presentation of vaccine-encoded antigens and activating antigen-specific CD8^+^ T cells. It is unclear if this CD8^+^ T cell bias can also be translated into similar finding in macaques or humans. In fact, an MVA vector with four deletions in different VACV immunomodulatory genes favors in macaques a CD4^+^ T cell response over a CD8^+^ T cell response [40]. However, we have previously described that MVA-B (with no further deletions) induced a preferential CD8^+^ T cell response in a prophylactic phase I clinical trial in humans [19], [20], suggesting that the observed effects of the double deletion mutant in mice might be translated to humans.

Interestingly, in the adaptive and memory responses, MVA-B ΔC6L and MVA-B ΔC6L/K7R induced a pattern with preference for GPN-specific CD8^+^ T cells, while MVA-B triggers a more distributed pattern of CD8^+^ T cells directed against Env, Gag and GPN. This shift towards a GPN-specific CD8^+^ T cell response by the deletion mutants might be related to the increased production of IFN-β, chemokine and pro-inflammatory cytokines promoted by the intracellular expression of GPN and/or by the lack of *C6L* and *K7R*. Thus, considering the biological role of *C6L* and *K7R* in blocking IFN signaling pathways, it can be suggested that the shift towards a GPN-response is the combined results of viral-induced IFN-β, chemokines and pro-inflammatory cytokines that stimulate T cell responses to the HIV-1 antigens and to improved functionality of DCs to serve as antigen presenting cells. Taking into consideration that during MVA-B infection gp120 is released from cells and GPN remains intracellular, and that infected DCs induced the expression of different immunomodulatory molecules [34], it will not be surprising if the deletion of viral genes acting as inhibitors of an inflammatory response might have a profound effect on the shift of immune responses, with enhanced GPN over Env response. Thus, differences in immunodominance are more likely due to the mode of action of *C6L* and *K7R* genes playing a regulatory role in the immune response. The functional relevance of GPN-specific CD8^+^ T cell response or Env-specific CD4^+^ T cell response in the efficacy of vaccination for prevention of HIV-1 infection remains unknown. More significantly, the T cell responses induced by all the immunization groups were maintained with time and the cell predominance was T effector memory (TEM) cells. Thus, in the memory phase MVA-B ΔC6L/K7R induced an increase in the percentage of HIV-1 specific CD8^+^ T effector cells. This T cell population has been shown to have powerful and direct antiviral capacity [41], [42], [43], [44], has been associated with HIV-1 viral control in early and chronic infection in humans [45], [46], [47], and on the early control of highly pathogenic SIV in non-human primates [48], [49]. Furthermore, these polyfunctional CD8^+^ T cells of effector memory phenotype are important for a protective immune response [44], [50].

In addition, all the vectors triggered anti-gp120 humoral responses, with MVA-B ΔC6L and MVA-B ΔC6L/K7R inducing slightly higher levels than MVA-B of anti-gp120 in the memory phase, although not significant. The durability of the B cell response may be mediated by enhanced levels of Env/Gag-Pol-Nef CD4 T-helper cells triggered by the vector. As suggested from the RV144 clinical trial, for vaccination to be effective the vector must induce antibody responses against Env, which might not need to be neutralizing but with binding affinity of the human antibodies for the V1V2 loops of gp120 [51]. While our studies have been done in mice, it is a positive response for an HIV/AIDS vaccine candidate that the improved vector MVA-B ΔC6L/K7R induced durable B and T cell responses specific for HIV-1 antigens.

A requirement for an HIV/AIDS vaccine candidate is the ability of a vector to activate innate and adaptive immune responses, to establish memory cells, expand the repertoire of epitopes recognized by T cells, and induce humoral responses to HIV-1 antigens. In fact, the vector MVA-B ΔC6L/K7R fulfill those requirements, triggering strong and polyfunctional CD4^+^ and CD8^+^ T cell responses against peptides spanning Env, Gag and Gag-Pol-Nef, and antibodies against HIV-1 gp120. Therefore, the results obtained here extend the previous results with MVA-B ΔC6L [15], and reinforce the need for an understanding on the role played by selected viral genes in the activation/suppression of immune responses, in order to improve the immunogenicity of poxvirus-based vaccine candidates.

VACV proteins C6 and K7 act intracellularly inhibiting IRF3/IFN-β signaling as confirmed here with the observation of up-regulation of IFN-β, TNFα and MIP-1α in human macrophages and moDC cells infected with MVA-B ΔC6L and MVA-B ΔC6L/K7R, being the double deletion mutant the most potent inducer. This IFN increase is likely to be biologically relevant, due to the important role of IFNs in innate control of HIV-1 replication blocking both early and late stages of the HIV-1 lifecycle ([52], [53], [54], [55], [56], [57], [58], for a review [59]). Moreover, it has been shown that IFN-β potently enhances immune responses *in vivo* through the stimulation of dendritic cells, acting as a link between the innate and the adaptive immunity [60], [61]. Furthermore, the co-induction of IFN-β and TNFα by MVA-B ΔC6L and MVA-B ΔC6L/K7R could represent an innate defense mechanism to restrict the vector replication in human cells, similar to what has been described with myxoma virus [62]. Nonetheless, the higher induction of IFN-β, TNFα and MIP-1α in human macrophages and moDC cells infected with MVA-B ΔC6L and MVA-B ΔC6L/K7R could explain the triggering of enhanced adaptive HIV-1 specific immune responses.

A number of MVA deletion mutants have been generated to date with the aim to define the contribution of one or more VACV genes in antigen specific immune responses. This has been tested in cells and in animal models, mice [14], [15], [39], [63], [64] and macaques [40], [65]. These studies have revealed that MVA with a single deletion of VACV genes encoding inhibitors of type 1 IFN signaling pathway (*C6L*; [15]), apoptosis (*F1L*; [39]), IL-18 binding protein (*C12L*; [63]) or a deletion of uracyl-DNA glycosylase gene (*UDG*, [65]), enhanced to various degrees the overall immune responses to HIV-1 antigens, evaluated by ELISPOT and/or ICS by analyzing the magnitude and polyfunctionality of CD4^+^ and CD8^+^ T cells that recognize specifically HIV-1 antigens. The responses against HIV-1 antigens were further enhanced by MVA vectors with two VACV gene deletions, *A41L*/*B16R* ([14] and *C6L*/*K7R*; this work) and also by NYVAC vectors with two deletions in VACV genes *B8R* and *B19R* [22]. However, when more gene deletions were introduced in the MVA vector the immune responses triggered by the vector were variable, either enhanced ([14], this work) or suppressed [40]. For example, four deletions in MVA [IL-18 binding protein (MVA008L; *C12L*), Toll/IL-1 receptor homolog (MVA159R; *A46R*), CC-chemokine binding protein (MVA153L; *B7R*) and secreted IL-1β receptor (MVA184R; *B16R*)], enhanced by six-fold the frequencies of HIV-1 specific CD4^+^ and CD8^+^ T cell responses, while an additional deletion of uracyl-DNA glycosylase (MVA101R) decreases the responses, as evaluated in macaques [40]. Moreover, Cottingham et al. described deletions in MVA of VACV genes *C12L*, *A44L* (hydroxysteroid dehydrogenase), *A46R* and *B7R* that had no effect on immune responses to viral antigens, while deletion of secreted IL-1β receptor (*B16R*) improves the responses, as defined in mice [64]. While direct comparison of results obtained with MVA from different laboratories are difficult to make, due to differences in vector generation, type and time of immune analysis, site of vector inoculation, dose of immunogen, and animal model, it is however a consistent finding for HIV-1 antigens, that single deletions of *C6L*, *C12L*, *F1L* or, double deletions of *A41L*/*B16R* or *C6L*/*K7R* (this work), and quadruple deletions of *C12L*+*A46R*+*B7R*+*B16R*, all showed a clear benefit to the MVA vector promoting enhanced immune responses to HIV-1 antigens.

Overall, deletion of VACV specific genes *C6L*/K7R from MVA blocking intracellularly the IFN signaling pathway triggered enhanced cellular immunogenicity against HIV-1 antigens in mice, demonstrating the role of these VACV genes as immunomodulators. The approach of targeting common pathways, like IRF3/IFN-β signaling, could be a general strategy to improve the immunogenicity of the MVA vector and of poxvirus-based vaccine candidates.

## Materials and Methods

### Ethics Statement

The animal studies were approved by the Ethical Committee of Animal Experimentation (CEEA-CNB) of Centro Nacional de Biotecnologia (CNB-CSIC, Madrid, Spain) in accordance with national and international guidelines and with the Royal Decree (RD 1201/2005). Permit numbers: 080030 and 11044.

Studies with peripheral blood mononuclear cells (PBMCs) from healthy blood donors recruited by the “Centro de Transfusión de la Comunidad de Madrid” (Madrid, Spain) were approved by the Ethical Committee of Centro de Transfusión de la Comunidad de Madrid (Madrid, Spain). Written informed consent was obtained from each donor before blood collection, for the purpose of this investigation according to a collaborative agreement between the “Centro de Transfusión de la Comunidad de Madrid” and the CNB-CSIC. All information was kept confidential.

### Cells and viruses

DF-1 cells (a spontaneously immortalized chicken embryo fibroblast cell line. ATCC, Manassas, VA) and primary chicken embryo fibroblast cells (CEF) [16] were grown in Dulbeccós modified Eaglés medium (DMEM) supplemented with 10% fetal calf serum (FCS). The human monocytic THP-1 cell line (ATCC, Manassas, VA) was cultured in RPMI 1640 medium containing 2 mM L-glutamine, 50 µM 2-mercaptoethanol, 100 IU/ml penicillin, 100 µg/ml streptomycin (complete medium; all from Invitrogen, San Diego, CA) and 10% heat-inactivated FCS (Sigma-Aldrich, St. Louis, MO), as previously described [15], [66]. THP-1 cells were differentiated into macrophages by treatment with 0.5 mM phorbol 12-myristate 13-acetate (PMA, Sigma-Aldrich) for 24 h before usage. PBMCs from buffy coats of healthy donors (recruited by the Centro de Transfusión de la Comunidad de Madrid, Madrid, Spain) were obtained by Ficoll gradient separation on Ficoll-Paque (GE Healthcare). Then, CD14^+^ monocytes were purified by depletion using Dynabeads® Untouched™ human monocytes kit (Invitrogen Dynal AS, Oslo, Norway), following the manufactureŕs protocol. To obtain moDCs, purified monocytes were cultured for 7 days in 6-well plates (3 × 10^6^ cells/well at 1×10^6^ cells/ml) in complete RPMI 1640 medium containing 10% heat-inactivated FCS and supplemented with 50 ng/ml granulocyte-macrophage colony-stimulating factor (GM-CSF) and 20 ng/ml IL-4 (both from Gibco-Life Technologies). Cell cultures were maintained at 37°C (CEF, THP-1 cells and moDCs) or 39°C (DF-1) in a humidified incubator containing 5% CO_2_. Cell lines were infected with viruses as previously described [16], [66].

The poxvirus strains used in this work included the attenuated modified vaccinia virus Ankara (MVA-WT) and the recombinant MVA-B expressing the HIV-1_BX08_ gp120 protein, as a cell-released product, and HIV-1_IIIB_ Gag-Pol-Nef as an intracellular polyprotein from HIV-1 clade B isolates [16]. MVA-B was used as the parental vector for the generation of the different deletion mutants. All viruses were grown in primary CEF cells, purified by centrifugation through two 36% (w/v) sucrose cushions in 10 mM Tris-HCl pH 9, and titrated in DF-1 cells by plaque immunostaining assay, using rabbit polyclonal antibody against VACV strain WR (Centro Nacional de Biotecnología; diluted 1:1000), followed by an anti-rabbit horseradish peroxidase (HRP)-conjugated secondary antibody (Sigma; diluted 1:1000), as previously described [67]. The titer determinations of the different viruses were performed at least three times. All viruses were free of contamination with mycoplasma or bacteria.

### Construction of plasmid transfer vectors pGem-RG-C6L wm and pGem-RG-K7R wm

The plasmid transfer vectors pGem-RG-C6L wm and pGem-RG-K7R wm were used for the construction of the recombinant viruses MVA-B ΔC6L and MVA-B ΔC6L/K7R, respectively, with *C6L* (*C6L* in Copenhagen strain of VACV is equivalent to *MVA 019L* in MVA) and *K7R* (*K7R* in Copenhagen strain of VACV is equivalent to *MVA 028R* in MVA) genes deleted (for simplicity, we used throughout the work the open reading frame nomenclature of Copenhagen strain to refer the MVA genes). pGem-RG-C6L wm generation was described previously [15]. pGem-RG-K7R wm was obtained by sequential cloning of *K7R* flanking sequences into the plasmid pGem-RG wm (4540 bp), whose generation was previously described [14], and contains dsRed2 and rsGFP genes under the control of the synthetic early/late (E/L) promoter. MVA-B genome was used as the template to amplify the left flank of *K7R* gene (358 bp) with oligonucleotides LFK7R-AatII-F (5′-CAAAGGACGTCATCATCATTTTTTCACC-3′) (AatII site underlined) and LFK7R-XbaI-R (5′-GCTCTAGAGAGACTATCTCACACAAAAG-3′) (XbaI site underlined). The left flank was digested with AatII and XbaI and cloned into plasmid pGem-RG wm, previously digested with the same restriction enzymes, to generate pGem-RG-LFsK7R wm (4865 bp). The repeated left flank of *K7R* gene (366 bp) was amplified by PCR from MVA-B genome with oligonucleotides LF´K7R-EcoRI-F (5′-CGGAATTCATCATCATTTTTTCACC-3′) (EcoRI site underlined) and LF´K7R-ClaI-R (5′-CCATCGATGACTATCTCACAAAAG-3′) (ClaI site underlined), digested with EcoRI and ClaI, and inserted into the EcoRI/ClaI-digested pGem-RG-LFsK7R wm to generate pGem-RG-LFdK7R wm (5190 bp). The right flank of *K7R* gene (366 bp) was amplified by PCR from MVA-B genome with oligonucleotides RFK7R-ClaI-F (5′-CCATCGATTCTAGAAAAAAAATTGAATTG-3′) (ClaI site underlined) and RFK7R-BamHI-R (5′-CGGGATCCAACAAGGGGTTGG-3′) (BamHI site underlined), digested with ClaI and BamHI and inserted into the ClaI/BamHI-digested pGem-RG-LFdK7R wm. The resulting plasmid pGem-RG-K7R wm (5526 bp) was confirmed by DNA sequence analysis and directs the deletion of *K7R* gene from MVA-B ΔC6L genome.

### Construction of MVA-B deletion mutants (MVA-B ΔC6L and MVA-B ΔC6L/K7R)

MVA-B deletion mutants generated in this study included MVA-B ΔC6L and MVA-B ΔC6L/K7R, with *C6L* and *C6L*-*K7R* VACV genes deleted, respectively. MVA-B deletion mutants were constructed by screening for transient Red2/GFP co-expression using dsRed2 and rsGFP genes as the transiently selectable markers, as previously described [14], [15]. After 6 consecutive rounds of plaque purification in DF-1 cells, MVA-B deletion mutants were obtained. The construction of the deletion mutant MVA-B ΔC6L has been previously described [15]. The double deletion mutant MVA-B ΔC6L/K7R was constructed using MVA-B ΔC6L as the parental virus and pGem-RG-K7R wm as the plasmid transfer vector.

### PCR analysis of MVA-B deletion mutants

To test the correct generation and purity of the MVA-B deletion mutants, viral DNA was extracted from DF-1 cells mock-infected or infected at 2 PFU/cell with MVA-WT, MVA-B, MVA-B ΔC6L or MVA-B ΔC6L/K7R, as previously described [16]. Primers RFC6L-AatII-F and LFC6L-BamHI-R, LFK7R-AatII-F and RFK7R-BamHI-R (described above), spanning *C6L and K7R* flanking regions, respectively, were used for PCR analysis of *C6L* and *K7R* loci. The amplification protocol was previously described [14]. PCR products were resolved in 1% agarose gel and visualized by SYBR Safe staining (Invitrogen). The *C6L* and *K7R* deletions were also confirmed by DNA sequence analysis.

### Expression of HIV-1_BX08_ gp120 and HIV-1_IIIB_ Gag-Pol-Nef proteins by MVA-B deletion mutants

To test the correct expression of HIV-1_BX08_ gp120 and HIV-1_IIIB_ Gag-Pol-Nef (GPN) proteins by MVA-B deletion mutants, monolayers of DF-1 cells were mock-infected or infected at 2 PFU/cell with MVA-WT, MVA-B, MVA-B ΔC6L or MVA-B ΔC6L/K7R. At 24 h post-infection, cells were lysed in Laemmli buffer, cells extracts were fractionated in 8% SDS-PAGE and analyzed by Western blot using rabbit polyclonal anti-gp120 antibody against IIIB (Centro Nacional de Biotecnología; diluted 1:3000) or polyclonal anti-gag p24 serum (ARP 432, NIBSC, Centralised Facility for AIDS reagent, UK; diluted 1:1000) to evaluate the expression of gp120 and GPN proteins, respectively. An anti-rabbit HRP-conjugated antibody (Sigma; diluted 1:5000) was used as secondary antibody. The immunocomplexes were detected using an enhanced HRP-luminol chemiluminescence system (ECL Plus; GE Healthcare).

### Analysis of virus growth

To determine virus-growth profiles, monolayers of DF-1 cells grown in 12-well tissue culture plates were infected in duplicate at 0.01 PFU/cell with MVA-B, MVA-B ΔC6L or MVA-B ΔC6L/K7R. Following virus adsorption for 60 min at 37°C, the inoculum was removed. The infected cells were washed once with DMEM without serum and incubated with fresh DMEM containing 2% FCS at 37°C in a 5% CO_2_ atmosphere. At different times post-infection (0, 24, 48 and 72 hours), cells were collected, freeze-thawed three times and briefly sonicated. Virus titers in cell lysates were determined by immunostaining as described above.

### RNA analysis by quantitative real-time polymerase chain reaction

Total RNA was isolated from THP-1 cells and moDCs mock-infected or infected with MVA-WT, MVA-B, MVA-B ΔC6L or MVA-B ΔC6L/K7R, using the RNeasy kit (Qiagen GmbH, Hilden, Germany). Reverse transcription of 500 ng of RNA was performed using the QuantiTect Reverse Transcription kit (Qiagen GmbH, Hilden, Germany). Quantitative PCR was performed with a 7500 Real-Time PCR System (Applied Biosystems) using the Power SYBR Green PCR Master Mix (Applied Biosystems), as previously described [66]. Expression levels of *IFN-β, IFIT1, IFIT2, MIP-1α, and HPRT* genes were analyzed by real-time PCR using specific oligonucleotides (sequence will be provided upon request). Gene specific expression was expressed relative to the expression of *HPRT* in arbitrary units (A.U.). All samples were tested in duplicates.

### IFN-β, TNF-α and MIP-1α measurements

IFN-β concentrations in cell-culture supernatants from moDCs mock-infected or infected with MVA-WT, MVA-B, MVA-B ΔC6L or MVA-B ΔC6L/K7R, were measured by ELISA (PBL Biomedical Laboratories, Picataway, NJ), following the manufactureŕs protocol. TNF-α and MIP-1α concentrations in cell-culture supernatants from moDCs mock-infected or infected with MVA-WT, MVA-B, MVA-B ΔC6L or MVA-B ΔC6L/K7R, were measured by Luminex assay.

### DNA vectors

The two DNA constructs expressing HIV-1_BX08_ gp120 (pCMV-_BX08_gp120) and HIV-1_IIIB_ Gag-Pol-Nef fusion protein (pcDNA-_IIIB_GPN) have been previously described [16]. Plasmids were purified using EndoFree plasmid Mega kit (Qiagen, Hilden, Germany), following manufactureŕs protocol, and diluted for injection in endotoxin-free phosphate-buffered saline (PBS).

### Peptides

HIV-1 peptide pools were provided by the EuroVacc Foundation and were previously described [16]. The peptides encompassed the Env, Gag, Pol, and Nef proteins of HIV-1 and were designed based on the sequence of the immunogens expressed by MVA-B. They spanned the HIV-1 Env, Gag, Pol and Nef regions from clade B included in the immunogens as consecutive 15-mers overlapping by 11 amino acids. The HIV-1_BX08_ gp120 protein was spanned by the Env-1 and Env-2 pools. The HIV-1_IIIB_ Gag-Pol-Nef fusion protein was spanned by the following pools: Gag-1, Gag-2, GPN-1, GPN-2, GPN-3 and GPN-4. The size and number of peptides included in each pool was previously described [14]. For immunological analysis, we grouped the peptides in three main pools: Env, Gag and GPN. The Env-pool comprises Env-1 + Env-2; Gag-pool comprises Gag-1 + Gag-2; and GPN-pool comprises GPN-1 + GPN-2 + GPN-3 + GPN-4.

### Mice immunization schedule

Female BALB/c mice (6–8 weeks old) were purchased from Harlan and stored at a pathogen-free barrier area of the CNB, in accordance to the recommendations of the Federation of European Laboratory Animal Science Associations. A DNA prime/MVA boost immunization protocol was performed as previously described [14], [16], to assay the immunogenicity of the different deletion mutants. Groups of animals (n = 8) received 100 µg of DNA-B (50 µg of pCMV-_BX08_gp120+50 µg of pCDNA-_IIIB_GPN) by intramuscular (i.m.) route and two weeks later received an intraperitoneal (i.p.) inoculation of 1×10^7^ PFU of the corresponding recombinant vaccinia viruses (MVA-B, MVA-B ΔC6L or MVA-B ΔC6L/K7R) in 200 µl of PBS. Mice primed with sham DNA (DNA-φ) and boosted by the nonrecombinant MVA-WT were used as a control group. At 10 and 52 days after the last immunization, 4 mice in each group were sacrificed using carbon dioxide (CO_2_), and spleens were processed to measure the adaptive and memory immune responses against HIV-1 antigens, respectively, by intracellular cytokine staining (ICS) assay. Three independent experiments have been performed.

### Intracellular Cytokine Staining (ICS) assay

The magnitude, polyfunctionality and phenotypes of the HIV-1-specific T cell adaptive and memory responses were analyzed by ICS, as previously described [14], [15]. After an overnight rest, 5×10^6^ splenocytes (depleted of red blood cells) were resuspended in RPMI 1640 supplemented with 10% FCS and containing 1 µl/ml Golgiplug (BD Biosciences) to inhibit cytokine secretion. Cells were seeded on M96 plates and stimulated for 6 h with HIV-1 Env-, Gag- or GPN-pools of peptides (5 µg/ml). Then, cells were washed, stained for the surface markers, fixed and permeabilized (Cytofix/Cytoperm kit; BD Biosciences), and stained intracellularly with the appropriate fluorochromes. For functional analyses, the following fluorochrome-conjugated antibodies were used: CD4-Alexa 700, CD8-V500, IFN-γ-PE-Cy7, TNF-α-PE and IL-2-APC. In addition, for phenotypic analyses the following antibodies were used: CD62L-FITC and CD127-PerCP-Cy5.5. All antibodies were from BD Biosciences. Dead cells were excluded using the violet LIVE/DEAD stain kit (Invitrogen). Cells were acquired using a LSRII flow cytometer (BD Immunocytometry Systems). Analyses of the data were performed using the FlowJo software version 8.5.3 (Tree Star, Ashland, OR). The number of lymphocyte-gated events ranged between 10^5^ and 10^6^. After gating, boolean combinations of single functional gates were then created using FlowJo software to determine the frequency of each response based on all possible combinations of cytokine expression or all possible combinations of differentiation marker expression. Background responses detected in negative-control samples were subtracted from those detected in stimulated samples for every specific functional combination.

### Antibody measurements by ELISA

Antibodies anti-HIV-1 gp120 MN envelope protein in serum from immunized mice were measured by ELISA as previously described [14], [16].

### Statistical procedures

The statistical analysis of ICS assay was realized as previously described [14], [68], using a novel approach that corrects measurements for the medium response (RPMI), calculating confidence intervals and p-values. Only antigen responses values significantly larger than the corresponding RPMI are represented. Background values were subtracted from all values used to allow analysis of proportionate representation of responses.
